# The Impact of the COVID-19 Pandemic on Loneliness Among University Students: A Cross-Sectional Study in Jordan

**DOI:** 10.7759/cureus.44013

**Published:** 2023-08-23

**Authors:** Odate K Tadros, Shereen Arabiyat, Deema Jaber, Mustafa Elayyan, Rewa Alawwa, Husam ALSalamat

**Affiliations:** 1 Department of Health Allied Sciences, Al-Balqa Applied University, Al-Salt, JOR; 2 Department of Biopharmaceutics and Clinical Pharmacy, School of Pharmacy, Zarqa University, Zarqa, JOR; 3 Department of Internal Medicine, Faculty of Medicine, Al-Balqa Applied University, Al-Salt, JOR; 4 Department of Biopharmaceutics and Clinical Pharmacy, School of Pharmacy, The University of Jordan, Amman, JOR

**Keywords:** jordan, mental health, university students, gender, loneliness, covid-19

## Abstract

Introduction: Loneliness is characterized by a sense of melancholy, emptiness, and despair, as well as a higher risk of both psychological and physical problems. Numerous post-coronavirus disease 2019 (COVID-19) sequela, both physically and mentally, have been caused by the global COVID-19 pandemic that has affected many people. Memory issues and loneliness were found to be uniquely correlated. The purpose of this study was to evaluate how the COVID-19 epidemic affected the levels of loneliness among Jordanian university students.

Methods: This is a cross-sectional questionnaire-based study. Google Forms were used for data collection, utilizing a three-item loneliness scale developed by the University of California, Los Angeles (UCLA), United States, which accounts for the following items: (1) how often does the participant feel about lacking companionship, (2) how often does the participant feel being left out, and (3) how often does the participant feel isolated from others. Each item was marked for three frequency levels of experience: (1) hardly ever, (2) some of the time, and (3) often. Responses to the three loneliness questions were graded on a scale of 3-9, with scores between 3 and 5 indicating "not lonely" and scores between 6 and 9 indicating "lonely."

Results: The study included 802 participating students. Specifically, 75.4% of the participants were females, 58% were aged 20-25, and 39% were 17-19. In addition, 37% were from the capital city, while 28% lived in the middle area. Seventy nine percent were from public universities, and 74% were studying in their second year. According to UCLA's three-item loneliness scale, 411 (51.2%) participants were lonely, while 391 (48.8%) participants were not feeling lonely. According to direct question answers, 576 (71.8%) participants were lonely, and 226 (28.2%) were not lonely.

Conclusion: The study concluded that the frequency of loneliness was high among Jordanian university students. However, both genders experienced equal levels of loneliness, while younger participants felt more lonely than older ones.

## Introduction

Loneliness is a complex emotional state characterized by feelings of sadness, emptiness, and despair, often accompanied by an increased risk of psychological and physical complications [1,2]. It can arise from various factors, such as stress, social separation, bereavement, and impaired social skills, among others [1]. The effects of loneliness are severe, encompassing adverse effects on mental health, such as anxiety and depression, as well as physical health issues, such as cognitive decline, poor sleep quality, and heightened susceptibility to cardiovascular diseases [3-5]. Although voluntary seclusion is very different [6], young individuals aged 16-24 years are particularly susceptible to loneliness, with a 27% higher likelihood of reporting feelings of loneliness compared to those aged 75 years or older [7].

Notably, loneliness has been associated with education and memory complaints [8]. University students, in particular, have been the focus of loneliness research for decades [9-12]. Studies have indicated that over 35% of university students experience loneliness, which is positively correlated with depression and anxiety [13]. Moreover, the coronavirus disease 2019 (COVID-19) pandemic exacerbated feelings of loneliness among young individuals, as reported in Italy, Germany, and the United States [14-16].

The impact of the COVID-19 pandemic on loneliness has been profound, with lockdowns, physical separation, and the shift to remote work and online education, altering the quality and quantity of social interactions worldwide [17,18]. As a result, loneliness rates have surged across the globe [18]. Against this backdrop, it is crucial to examine how the COVID-19 epidemic has affected loneliness levels among university students in Jordan.

The purpose of this study was to evaluate how the COVID-19 epidemic affected the levels of loneliness among Jordanian university students. By investigating this aspect, the research aimed to provide valuable insights into the psychosocial consequences of the pandemic on young adults in the educational setting and contribute to potential interventions and support measures to address the issue of loneliness during such challenging times.

## Materials and methods

Study design

**Figure 1:**
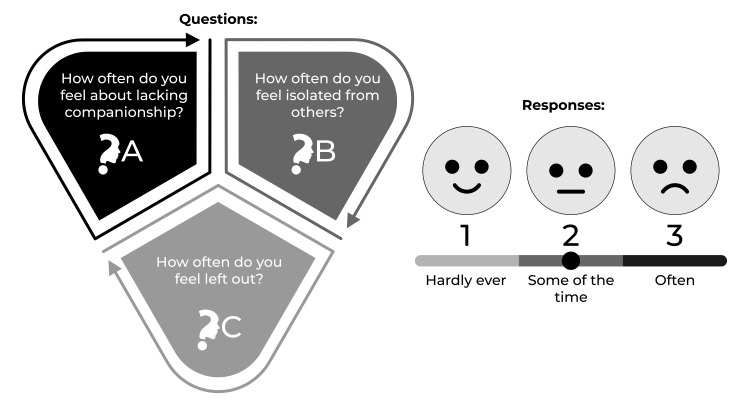
Three-item loneliness scale. In the three-item loneliness scale [2,19], the participants were asked about their feelings on the represented aspects of their lives. The frequency of each question and response was recorded. Scores between 3 and 5 indicate "not lonely," and scores between 6 and 9 indicate "lonely." According to UCLA, the original publishers, "students and researchers do not need permission to use the UCLA Loneliness Scale for non-profit research purposes may do so without permission."

This research employed a cross-sectional survey-based approach using Google Forms to gather data on demographics and measure loneliness using the University of California, Los Angeles (UCLA) three-item loneliness scale [2,19] (refer to Figure 1). The scale includes a direct question about loneliness: "Do you feel lonely?" Responses to the three-item loneliness questions were scored on a scale of 3-9, with scores between 3 and 5 indicating "not lonely" and scores between 6 and 9 indicating "lonely" [20]. According to UCLA, the original publishers, "students and researchers do not need permission to use the UCLA Loneliness Scale for non-profit research purposes may do so without permission."

Sample size calculation

In this research, and according to the second semester 2020-2021 general statistical report for Jordanian universities [21], our estimated population consisted of approximately 300,000 students in both public and private universities across all undergraduate academic years, attending both public and private universities. We performed a sample size calculation, estimating that around 400 samples were needed for our survey in order to obtain a 95% confidence level with a 5% margin of error. We may confidently assert that there is a 95% probability that the true value within the population falls within 5% of the measured value received from the survey by gathering 400 or more randomly selected surveys. We meticulously applied random sample approaches to assure the validity and reliability of our findings. This substantially reduced potential bias and allowed us to reach significant conclusions that are applicable to the larger community of university students.

Ethical consideration

This investigation adhered to the World Medical Association's Declaration of Helsinki. This study's execution was given approval by the institutional review board (IRB) committee of Al-Balqa Applied University (Approval No. 26/3/1/176). Participants gave informed consent and enrolled voluntarily. Participants were given a thorough description of the study's goals before beginning the questionnaire, with a focus on privacy protection. Participants' names and email addresses were not gathered, and neither were any other personal data. They were specifically asked if they wanted to take part in the questionnaire, and those who did were given the option to opt-out at any time throughout the process. The study's data's security and confidentiality were prioritized, and the researchers were given strict access only.

Data collection

Data collection occurred between July and September 2020, during the pandemic. The survey was conducted in Arabic in a context similar to previous research [22], and participants were asked to provide consent to ensure the confidentiality of their responses. The survey was promoted through social media platforms such as Facebook and WhatsApp. The study targeted undergraduate university students of all ages attending public or private universities in Jordan.

Data analysis

The collected data were coded and analyzed using Microsoft Office (MS Excel, version 2016) and the Statistical Package for Social Sciences (SPSS, version 27; IBM SPSS Statistics for Windows, Armonk, NY). Descriptive analysis was performed using an independent variable t-test, one-way analysis of variance (ANOVA), and post hoc tests. Significance was assessed using p-values (p < 0.05). The internal consistency of the data was evaluated for reliability analysis using Cronbach's alpha (α), a measurement that determines the internal consistency of an assessment instrument. It is considered appropriate for Cronbach's alpha to be between 0.6 and 0.8 [23].

## Results

Descriptive analysis

A total of 802 participants took part in this study, comprising both male and female students. More than half of the participants fell within the age range of 20-25 years, accounting for 58.0% of the sample. Among the respondents, the highest proportion hailed from Amman, the capital city of Jordan, representing 36.7% of the total. Furthermore, a significant portion of the students attended public universities, accounting for 78.7% of the sample.

Table 1 presents a detailed breakdown of the sample characteristics, including age, gender, place of residence, type and location of universities, academic year, and educational level, expressed in both frequency and percentage terms.

**Table 1:** Demographic data of participants (N=802).

Variables	Group	N (%)
Gender		
	Male	197 (24.6)
	Female	605 (75.4)
Age Groups		
	17–19	310 (38.7)
	20–25	465 (58)
	26–30	15 (1.9)
	31–40	8 (1.0)
	> 40	4 (0.5)
Residence		
	Capital City	294 (36.7)
	Middle Area	228 (28.4)
	North	156 (19.5)
	South	124 (15.5)
Academic Year		
	First Year	149 (18.6)
	Second Year	592 (73.8)
	Third Year	39 (4.9)
	Fourth Year	16 (2.0)
	Fifth Year	3 (0.4)
	More than Fifth Year	3 (0.4)
University Type		
	Private University	171 (21.3)
	Public University	631 (78.7)

Reliability statistics

Cronbach's alpha was used to assess the data's internal consistency, and the result was 0.69. Given that there are nine factors in the data, this result indicates a fair degree of internal consistency.

Factor analysis

Table 2 displays the variance (extraction) for each factor, as well as the commonalities. The observed values are all greater than 0.5, indicating substantial variance explained by the factors, except for gender, which exhibits the lowest value.

**Table 2:** Factor extraction. Extraction Method: Principal Component Analysis.

Factor Extraction	Extraction
Gender	0.133
Residence	0.648
Academic year	0.552
University type	0.612
Feel left out	0.956
Lack companionship	0.951
Feel isolated	0.959
Loneliness from scale	1.000
Direct question	0.505

The data were subjected to analysis based on the initial eigenvalues, and the sums of squared loadings for the extracted components were calculated. Variables associated with eigenvalues greater than 1 were chosen as relevant components. Table 3 presents the results, indicating that the first component has a value of 3.885 (>1), the second component has a value of 1.1406 (>1), and the third component has a value of 1.024 (>1). Furthermore, the extracted sum of squares reveals that the first factor accounts for 43.169% of the variance, the second factor explains 15.625%, and the third factor explains 11.381%. As a result, the data were summarized into three components, representing the association between loneliness and participants' demographic characteristics, as well as the results of frequency analysis.

**Table 3:** Loading extractions of the variables required for evaluation. Extraction Method: Principal Component Analysis. NA: Not Applicable.

Component	Initial Eigenvalues			Extraction Sums of Squared Loadings		
	Total	% of Variance	Cumulative %	Total	% of Variance	Cumulative %
1	3.885	43.169	43.169	3.885	43.169	43.169
2	1.406	15.625	58.794	1.406	15.625	58.794
3	1.024	11.381	70.175	1.024	11.381	70.175
4	0.981	10.898	81.073	<1.00	NA	NA
5	0.960	10.663	91.736	<1.00	NA	NA
6	0.611	6.791	98.527	<1.00	NA	NA
7	0.074	0.823	99.350	<1.00	NA	NA
8	0.058	0.650	100.000	<1.00	NA	NA
9	-5.052E-15	-5.613E-14	100.000	<1.00	NA	NA

Frequency analysis

Table 4 presents the frequency distribution of loneliness scores based on the three-grade scale. Among the participants, 51.2% scored between 3 and 5, while 48.8% scored between 6 and 9. Additionally, when assessing loneliness through direct questions, 71.8% of the participants reported feeling lonely, while 28.2% indicated not feeling lonely. The findings indicate that loneliness scores within the 3-5 range were more prevalent compared to scores in the 6-9 range. Moreover, the direct question on loneliness revealed that nearly three-quarters of all participants experienced feelings of loneliness.

**Table 4:** Frequency of loneliness among study participants (N=802).

Question	Answer	N (%)
How often do you feel a lack of companionship?		
	Hardly ever	409 (51.0)
	Some of the time	331 (41.3)
	Often	62 (7.7)
How often do you feel left out?		
	Hardly ever	413 (51.5)
	Some of the time	326 (40.6)
	Often	63 (7.9)
How often do you feel isolated from others?		
	Hardly ever	407 (50.7)
	Some of the time	336 (41.9)
	Often	59 (7.4)
Loneliness from the scale?		
	3–5	411 (51.2)
	6–9	391 (48.8)
Do you feel lonely (direct question)?		
	Yes	576 (71.8)
	No	226 (28.2)

Association between loneliness and demographic characteristics of participants

The analysis of the association between loneliness and demographic parameters revealed no significant correlation through direct questions. However, a notable association was found between loneliness and certain demographic factors, including age, residence area, and academic year. Post-hoc analysis further elucidated the specific associations: participants residing in the capital city of Amman exhibited higher levels of loneliness compared to those living in other areas (p-value = 0.006). Additionally, students in their second academic year reported greater loneliness compared to students in other academic years (p-value = 0.015). Furthermore, participants aged 17-19 experienced higher levels of loneliness compared to other age groups, as indicated in Table 5.

**Table 5:** Association between loneliness and demographic characteristics of study participants (N=802). P-values from chi-square are considered significant if < 0.05. *Significant values.

Demographic parameter	Loneliness	P-values
Gender		
	Direct question	0.860
	Scale calculated	0.098
Age		
	Direct question	0.505
	Scale calculated	0.023^*^
Residence area		
	Direct question	0.436
	Scale calculated	0.006^*^
Academic year		
	Direct question	0.423
	Scale calculated	0.015^*^
Public or private university		
	Direct question	0.398
	Scale calculated	0.574

## Discussion

The present study investigated loneliness levels among Jordanian university students, utilizing both a three-grade scale and direct questions on demographic factors, such as gender, age, residence area, academic year, and university type. The findings revealed that nearly three-quarters of the participants reported feeling lonely. Previous studies followed a similar approach [24,25] to determine loneliness levels. Interestingly, there was no significant difference in loneliness between males and females, contrary to previous studies that suggested females may be more prone to loneliness [26]. However, other types of research conducted in Latin America and the Caribbean indicated higher loneliness rates among girls compared to boys [27]. On the contrary, a different study reported that men may experience loneliness more intensely due to societal expectations [28].

Age emerged as a significant factor affecting loneliness, in line with prior research [29]. The current study found that younger students aged 17-19 experienced higher levels of loneliness compared to their older counterparts. This trend aligns with previous findings that suggested younger individuals may feel lonelier than middle-aged individuals, who in turn may experience more loneliness compared to older individuals [29].

Furthermore, participants living in Jordan's capital city, Amman, exhibited significantly higher levels of loneliness compared to those in other regions. This observation corresponds to studies highlighting that capital cities often foster loneliness due to overcrowded environments and limited exposure to green spaces, as exposure to green environments and overcrowded places can alleviate loneliness, limited exposure will increase loneliness risk [28]. The busyness and isolated nature of life in capital cities may contribute to increased feelings of loneliness [29].

Additionally, the study found that second-year students experienced higher loneliness levels compared to other academic year groups. This finding may be attributed to the impact of the COVID-19 pandemic, which led to delayed social interactions for first-year students, who faced challenges in building friendships due to lockdown measures and online learning. In contrast, older students already had established friendships from on-campus education, potentially contributing to lower loneliness levels [30]. Overall, the COVID-19 pandemic likely exacerbated loneliness among university students.

Limitations

Several limitations need to be acknowledged in this study. The online distribution of the questionnaire may have resulted in uneven responses from students in public and private universities, potentially affecting the representativeness of perspectives among Jordanian students. Moreover, clusters of students based on geographic location, type of university, and insufficient information about the kind of academic background of participating students may limit the generalizability of the findings to students in other settings, geographic locations, or academic disciplines. To enhance the comprehensiveness of future research, it is advisable to include a more balanced representation of universities and encompass an identifiable range of academic fields for a more comprehensive evaluation of student loneliness.

## Conclusions

This study revealed a significant prevalence of loneliness among Jordanian university students, ranging from 51% to 71%. These findings highlight the importance of recognizing university students as a vulnerable group, especially during the COVID-19 pandemic, which may have long-term psychosocial consequences. While no gender differences in loneliness were observed, age emerged as a contributing factor, with younger students experiencing higher loneliness levels. Additionally, living in densely populated areas with limited green spaces, such as capital cities, was associated with heightened loneliness. It is crucial to address and mitigate loneliness to prevent potential adverse physical and mental health consequences. This study calls for further research on loneliness in Jordan to better understand and address this challenging social affliction.
